# Trastuzumab deruxtecan: Defining a novel systemic treatment standard for HER2-positive breast cancer brain metastases?

**DOI:** 10.1093/neuonc/noae202

**Published:** 2024-10-03

**Authors:** Rupert Bartsch, Matthias Preusser

**Affiliations:** Division of Oncology, Department of Medicine 1, Medical University of Vienna, Vienna, Austria; Division of Oncology, Department of Medicine 1, Medical University of Vienna, Vienna, Austria

Brain metastases (BM) will occur in approximately half of all patients with HER2-positive metastatic breast cancer, causing a major increase in morbidity and mortality.^1^ In patients with limited intracranial disease and stable metastases in the extracranial compartment, stereotactic radiosurgery is preferred. But what about patients with parallel progression of intra- and extracranial lesions or in the case of extensive intracranial disease, when only whole-brain radiotherapy (WBRT) was possible? Systemic therapy of active (i.e. newly diagnosed or progressing) HER2-positive BM is well established today, with small-molecule tyrosine-kinase inhibitors (TKIs) leading the path. The latest iteration of this strategy—the triple combination of tucatinib, trastuzumab, and capecitabine (TTC)—is regarded as the standard of care based on the results of the HER2CLIMB trial, where a significant improvement in CNS progression-free survival (PFS) as well as overall survival was established in a population having received prior trastuzumab, pertuzumab, and trastuzumab emtansine (T-DM1).^2^ Therefore, the ASCO-SNO-ASTRO guidelines list TTC as the preferred brain-specific regimen and the EMSO guidelines recommend TTC as second-line and beyond therapy in a population with active BM without indication for immediate local intervention.^3,4^ Meanwhile, the antibody-drug conjugate (ADC) trastuzumab deruxtecan (T-DXd) was established as the novel second-line treatment standard in patients progressing on prior trastuzumab and pertuzumab, with an unprecedented disease control of approximately 2 years in the DESTINY-Breast03 trial.^5^ In addition, smaller phase II trials such as TUXEDO-1 and DEBBRAH suggested relevant intracranial activity as well, with response rate ranging from 45% to 70%.^6,7^ Furthermore, long-term outcomes of TUXEDO-1 and a joint analysis of 44 patients with untreated BM in the pivotal trials indicated excellent disease control.^8,9^ While these results leave little doubt regarding the activity of ADCs in BM in principle, the overall small patient numbers did not trigger any change in the guidelines to date.

At this year’s annual meeting of the European Society of Medical Oncology, primary results from the phase 3b/4 single-am open-label DESTINY-Breast12 trial were presented and simultaneously published in *Nature Medicine*.^10^ Overall, 504 patients with HER2-positive metastatic breast cancer were treated within a BM (*n* = 261; *n* = 106 active BM) and a non-BM cohort (*n* = 241), respectively. Nearly all patients had received prior trastuzumab and pertuzumab, with approximately 40% having received prior T-DM1 as well; prior tucatinib was not allowed. PFS was defined as the primary endpoint in the BM cohort. A 12-month PFS rate of 61.6% (95% CI 54.9-67.6) was reported with comparable results in the stable and active BM groups; in a post hoc analysis, median PFS was 17.3 months (95% CI 13.7-22.1). In 138 patients with measurable BM at baseline, the objective intracranial response rate (ORR) was 71.7%, with the numerically highest ORR in the subset of patients with previously untreated active lesions (82.6%).

In this patient population, no new safety signals were observed but interstitial lung disease (ILD) remains an issue: While DESTINY-Breast03 reported no fatal cases of ILD, there were nine fatalities in DESTINY-Breast12. Importantly, the majority of these cases were observed in the BM cohort (*n* = 6) with opportunistic co-infections occurring in 4 out of 6 patients. Despite the maximum daily dose of dexamethasone limited to 3 mg per day within the study, we assume that this observation may be linked to chronic corticosteroid use and therefore suggest appropriate microbiologic diagnostics in all patients with ILD. In addition, chemoprophylaxis in all T-DXd patients requiring corticosteroids for 4 weeks or longer should be considered irrespective of dose. Education of patients and site staff remains paramount to ensure early ILD detection and initiation of appropriate immunosuppressive therapy.

With ILD risk in mind, DESTINY-Breast12 clearly points to the fact that T-DXd has considerable intracranial activity, both in terms of response rate and disease control, making this an attractive option for BM patients. But does this single-arm trial provide sufficient evidence to support a systematic therapy first approach in general and the use of T-DXd in particular in patients with active BM? This question must be answered in the light of available evidence and the specific clinical situation. In cases where extracranial disease is stable and stereotactic radiotherapy to brain lesions is feasible, local treatment remains the preferred approach with continuation of the ongoing (and extracranially still active) systemic regimen. In case of parallel extracranial progression requiring a switch of systemic therapy and/or if WBRT was the only local treatment approach possible, T-DXd now appears to be the preferred option whenever indicated in principle (e.g. in the second-line setting). To allow for a complete appraisal of any drug in metastatic disease; however, patient-reported outcomes are required. Quality-of-life (QoL) data from DESTINY-Breast12 are yet pending but reassuringly, TUXEDO-1 has shown maintained global QoL and cognitive function over the entire treatment period.^8^

Therefore, results form DESTINY-Breast12 resonate in a large population the activity observed in previous smaller studies and therefore support the use of T-DXd whenever clinically indicated irrespective of the presence or absence of active BM. Tucatinib has a role in the next subsequent treatment line and the authors of HER2CLIMB must once again be commended for having conducted the largest randomized trial with regards to the number of patients with active BM to date. More commonly, an alternative approach is chosen in drug development with smaller phase II studies evaluating the potential activity of novel drugs in BM patients and the TUXEDO family of trials (TUXEDO-1, NCT04752059; TUXEDO-2, NCT05866432; TUXEDO-3, NCT05865990; TUXEDO-4, NCT06048718) is a noteworthy example. For the future, however, the authors of this editorial strongly suggest allowing patients with active BM onto pivotal trials to determine the effect of innovative agents as early as possible in what remains a clinically high-risk population.
